# Open access, open data and peer review

**DOI:** 10.1186/s13059-020-02005-3

**Published:** 2020-05-04

**Authors:** Jernej Ule

**Affiliations:** 1 The Francis Crick Institute, 1 Midland Road, London, NW1 1AT UK; 2 Department of Neuromuscular Diseases, UCL Queen Square Institute of Neurology, Queen Square, London, WC1N 3BG UK; 3 National Institute of Chemistry, Hajdrihova 19, SI-1001 Ljubljana, Slovenia

I am very fond of open access journals like *Genome Biology*. Another champion of such journals is Plan S, launched by Science Europe in 2018 and adopted by many funding agencies, which aims to encourage scientists to publish in open access journals or platforms [1]. I touch on some recent discussion of this topic and then highlight the need to link it to accessible raw and processed data associated with publications, which is particularly important in the genomics field.

In the companion editorial by Halffman and Horbach, editorial innovations are discussed that could make the peer review process more cost effective while retaining the quality of the process. In the age of information overload, with the large numbers of papers coming online each day, a high-quality peer review process remains of paramount importance, not only to evaluate scientific quality but also to identify the most ‘paradigm-shifting’ studies. However, the cost of this process increases with the selectivity of the journal: Springer Nature estimates that it costs, on average, €10,000–30,000 to publish an article in one of its journals, partly due to low acceptance rate and partly in order to produce non-research content, such as news and opinion articles [2]. Thus, a journal is not just a conduit for papers, but can help in organising knowledge exchange in a field in general, which is particularly the case for journals that attract transformative work. In order to be able to judge the transformative nature of submitted papers, editors from journals such as *Genome Biology* spend a considerable amount of time engaging with the scientific community in conferences and communicating with other editors in the team and with the editorial board. Thus, the article processing charges (APCs) of the top tier journals would increase if they were to switch to full open access, which could shift from inequity in access to published work to inequity in access to publishing, as scientists and their funders in emerging economies may be less capable of shouldering such APC costs [3, 4]. Moreover, society journals and certain fields such as chemistry and humanities are particularly reliant on income from subscriptions to complement their relatively low APC revenues, and if they were less able to adapt, the full open access model could increase the monopoly of large publishing houses who can more easily change their business models [5, 6].

In response to these concerns, Plan S recently adopted a more flexible stance towards hybrid open access and towards ‘new, innovative publishing models’ that would retain a diversity of cost-effective publishing options [7]. It has also been proposed that the costs of open access could be brought down if work of professional editors was largely replaced by academic editors, pioneered by journals such as *eLife*. This works as an excellent complementary system, but I wonder if we scientists could find sufficient time to act as editors for most scientific literature. The most brilliant scientists can be the worst editors if they have little time to read or engage with a paper, and we scientists have networks of colleagues and other biases of our own that are hard to avoid. The other alternative would be a system of public reviews such as PubPeer, twitter threads and science blogs, integrated with machine learning that would collate all information to rank the impact of papers. Again, while this is already developing into a valuable and ongoing complement that is bound to grow, it lacks moderation by editors and could bias towards those who invest more effort into networks or language of self-promotion as a way to stand out of the crowd if it were to replace peer review. Indeed, learning from the rapid rise of social networks over the last decade and their suddenly dominant role in news distribution demonstrates their potential for skewing the conversation into extremes.

A compromise that would satisfy most concerns has recently been proposed as a ‘Plan U’, in which funders require that grantees deposit manuscripts on a preprint server such as bioRxiv under a Creative Commons Attribution licence (CC BY) before submission and peer review in any type of journal [8]. While this sounds like a reasonable option, it leaves the question of open data unresolved. Currently, bioRxiv submissions are not obliged to provide the data that accompany the manuscript, so the underlying data are most often provided only as part of the final publication. This is creating a system where one can plant a flag in a research field without the need to provide the associated data that would be essential to evaluate the validity of conclusions. A further obstacle that is hampering our field is the rapid evolution of methods, data types and annotations, which are hard to properly curate when submitting to generic repositories such as GEO. For example, the study of protein-RNA interactions through a method called CLIP has already led to over 30 variant methods that differ aspects such as barcoding and crosslink site definition, and therefore, more specialised repositories are needed that enable method-specific annotation, quality control and analysis and provide both raw and processed data along with various visualisation tools [9]. Initiatives to support the development of new biomedical data resources are well placed to tackle such challenges [10], and publishers are looking for business models that could link new types of data repositories to the review process and to final publications [4]. It would make a great difference to reviewers to be able to explore processed data through standardised online platforms that provide method-specific metrics and visualisation.

In conclusion, the innovations in science publishing are taking place at multiple levels: open access to publications, presentation of data in an accessible manner, transparent and unbiased systems for evaluating scientific output, and reasonable costs of publishing, including in the top tier journals. It will be exciting to watch as new innovations emerge at all of these levels and to see journals such as *Genome Biology* find ways to link open access, new data curation and repository innovations with high-quality peer review and reasonable publication charges.
